# Expansion of *Pleioblastus amarus* in tea plantations significantly enhances the appearance and nutritional composition of bamboo shoots but adversely affects palatability

**DOI:** 10.1186/s12870-024-05856-1

**Published:** 2024-12-03

**Authors:** Lili Fan, Shuanglin Chen, Zongming Cai, Ziwu Guo, Jie Yang, Rong Zheng, Ruicai Hu

**Affiliations:** 1 Research Institute of Subtropical Forestry, Chinese Academy of Forestry, Hangzhou, 311400 China; 2 Experimental Center of Subtropical Forestry, Chinese Academy of Forestry, Xinyu, 336600 China; 3 Fujian Academy of Forestry Sciences, Fuzhou, 350012 China; 4 Longyou County Forestry Technology Extension Station, Quzhou, 324400 China

**Keywords:** Bamboo expansion, *Pleioblastus amarus*, Tea plantations, Bamboo shoot quality, Nutritional composition, Palatability

## Abstract

The expansion of *Pleioblastus amarus* into tea plantations introduces environmental heterogeneity, significantly influencing the growth and quality of bamboo shoots. This study examined the effects of bamboo expansion on the appearance, nutrition, and palatability of bamboo shoots, utilizing partial least squares structural equation modeling (PLS-SEM) to identify key influencing factors. Results revealed that bamboo expansion increased shoot diameter, length, and fresh weight, enhancing overall size and edibility, particularly in the tea-bamboo mixed forest center zone (TBC), where appearance quality peaked. Nutritional analysis revealed substantial increases in protein, fat, starch, and vitamin C content after bamboo expansion, along with the improvements in amino acid score (*AAS*), essential amino acid index (*EAAI*), and nutritional index (*NI*), indicating elevated nutritional value. However, despite the rise in soluble sugars and flavor-enhancing amino acids, higher levels of total acids, oxalic acid, tannins, and cellulose diminished the palatability, notably in TBC site. PLS-SEM further indicated that while bamboo expansion positively influenced shoot appearance and nutrition, soil factors predominantly drove these changes and concurrently detracted from overall palatability. These findings provide a framework for enhancing bamboo shoot quality and optimizing management practices in tea plantation ecosystems.

**Supplementary Information:**

The online version contains supplementary material available at 10.1186/s12870-024-05856-1.

## Introduction

Bamboo shoots, a significant edible product derived from bamboo species, play a crucial role in influencing consumer acceptance and determining market competitiveness [1]. Enhancing the quality of bamboo shoots is essential not only for the economic development of the bamboo shoot industry but also for maximizing the economic returns of bamboo forests [2, 3]. The quality of bamboo shoots is shaped by a complex interplay of genetic traits, environmental conditions, and cultivation practices [4], encompassing key attributes such as appearance, nutritional value, and palatability [5]. Among environmental factors, soil properties, climate, and water availability are particularly influential, directly or indirectly affecting the growth, development, and overall quality of bamboo shoots [6–8]. For example, variations in soil fertility can significantly alter the nutritional content and appearance of bamboo shoots [9, 10], while shifts in climate and water availability impact their growth cycle and palatability [11]. Human interventions, including fertilization, irrigation, and harvesting practices, further modify environmental conditions and cause variations in bamboo shoot quality [5, 6, 9, 12]. Therefore, understanding these environmental influences is critical for the production of high-quality bamboo shoots.

Bamboo, as a clonal plant, possesses an extensive underground rhizome system that facilitates its expansion into adjacent ecosystems under favorable conditions [13, 14]. This expansion often alters environmental factors such as light availability, nutrient levels, water supply, and spatial structure [4, 15], which in turn can significantly influence bamboo shoot quality. For example, variations in light intensity can affect photosynthetic efficiency [16], thereby influencing the accumulation of nutritional components in bamboo shoots. Similarly, changes in nutrient and water availability are closely linked to the growth rate and overall quality of the shoots [9, 10, 17]. Alterations in spatial structure, such as changes in stand density and distribution, also impact visual appeal and palatability of bamboo shoots [12, 18, 19]. Understanding how these environmental modifications associated with bamboo expansion affect shoot quality is essential for elucidating the underlying mechanisms and for developing targeted management strategies.

The tea-producing regions of Zhejiang and Fujian in China share similar environmental conditions with bamboo forests, creating an ideal setting for the natural expansion of bamboo into tea plantations [17, 20, 21]. This expansion is often facilitated by factors such as improper management practices, soil degradation, and insufficient pest control [17, 22], which contribute to the decline of tea plantations and create opportunities for bamboo colonization. Bamboo expansion modifies critical environmental variables, including light availability, nutrient levels, and spatial structure, which in turn affect both tea plants (*Camellia sinensis*) and bamboo shoots [21, 23–25]. However, research on bamboo-tea symbiosis has largely focused on the ecological benefits and tea production outcomes, such as enhanced soil quality, increased biodiversity, microclimate regulation, pest reduction, and economic returns [22, 25], with little attention given to how bamboo expansion impacts bamboo shoot quality. In particular, the effects on appearance, nutritional composition, and palatability of bamboo shoots has not been adequately explored, creating a research gap that this study aims to address.

*Pleioblastus amarus*, known for its resilience and adaptability, thrives in diverse natural environments and is highly competitive [26, 27]. In recent years, *P. amarus* has gained commercial attention for its high-quality bamboo shoots, valued for their appealing appearance, rich nutritional profile, and desirable culinary attributes such as tenderness, crispness, and juiciness [28, 29]. These qualities have made *P. amarus* a valuable species in the bamboo shoot market. The expansion of *P. amarus* into tea plantations presents a unique ecological interaction, potentially affecting bamboo shoot quality. While previous studies have highlighted environmental influences on bamboo shoot growth [9, 10, 12, 30, 31], the specific impacts of habitat heterogeneity within tea plantations remain unclear.

This study aims investigates the impact of *P. amarus* expansion on bamboo shoot quality in the expansion zone. By examining the growth dynamics of *P. amarus* and tea plants, alongside the soil properties at the interface, we seek to identify the key environmental drivers of bamboo shoot quality. The findings of this research are expected to provide valuable insights into the ecological interactions between bamboo-tea interactions and offer practical recommendations for the sustainable management and rehabilitation of declining tea plantations. The key questions addressed in this study are: (1) Do bamboo shoots exhibit significant quality differences across the expansion zone as *P. amarus* expands into tea plantations? (2) How do the growth characteristics of *P. amarus*, its competitive dynamics with tea plants, and soil properties influence bamboo shoot quality? (3) Does the expansion of *P. amarus* enhance its adaptability to the heterogeneous environments within tea plantations?

## Materials and methods

### Study site overview

The study was conducted in Muchen Township, Longyou County, Zhejiang Province, China (28°49′4.85″N, 119°13′25.88″E), a region characterized by a subtropical monsoon climate with distinct seasonal variations. The site receives an average annual precipitation of 1,620 mm, with a mean temperature of 17.40℃. The frost-free period extends for approximately 261 days annually, with an average relative humidity of 79%. The area enjoys around 1,769 h of sunshine per year. The soil at the site predominantly consists of sandy red soil, with a depth ranging from 70 to 100 cm.

The tea plantation under study was originally established in 1972 for green tea production. However, since 2008, the plantation has been progressively abandoned, transitioning from an intensive management regime to a more extensive, less managed state. This shift has facilitated the unimpeded expansion of the *P. amarus* forest into the former tea-growing areas. The expansion has resulted in approximately 1 hm^2^ of bamboo forest encroaching upon what is now an 0.53 hm^2^ remnant tea plantation.

### Experimental design

**Fig. 1:**
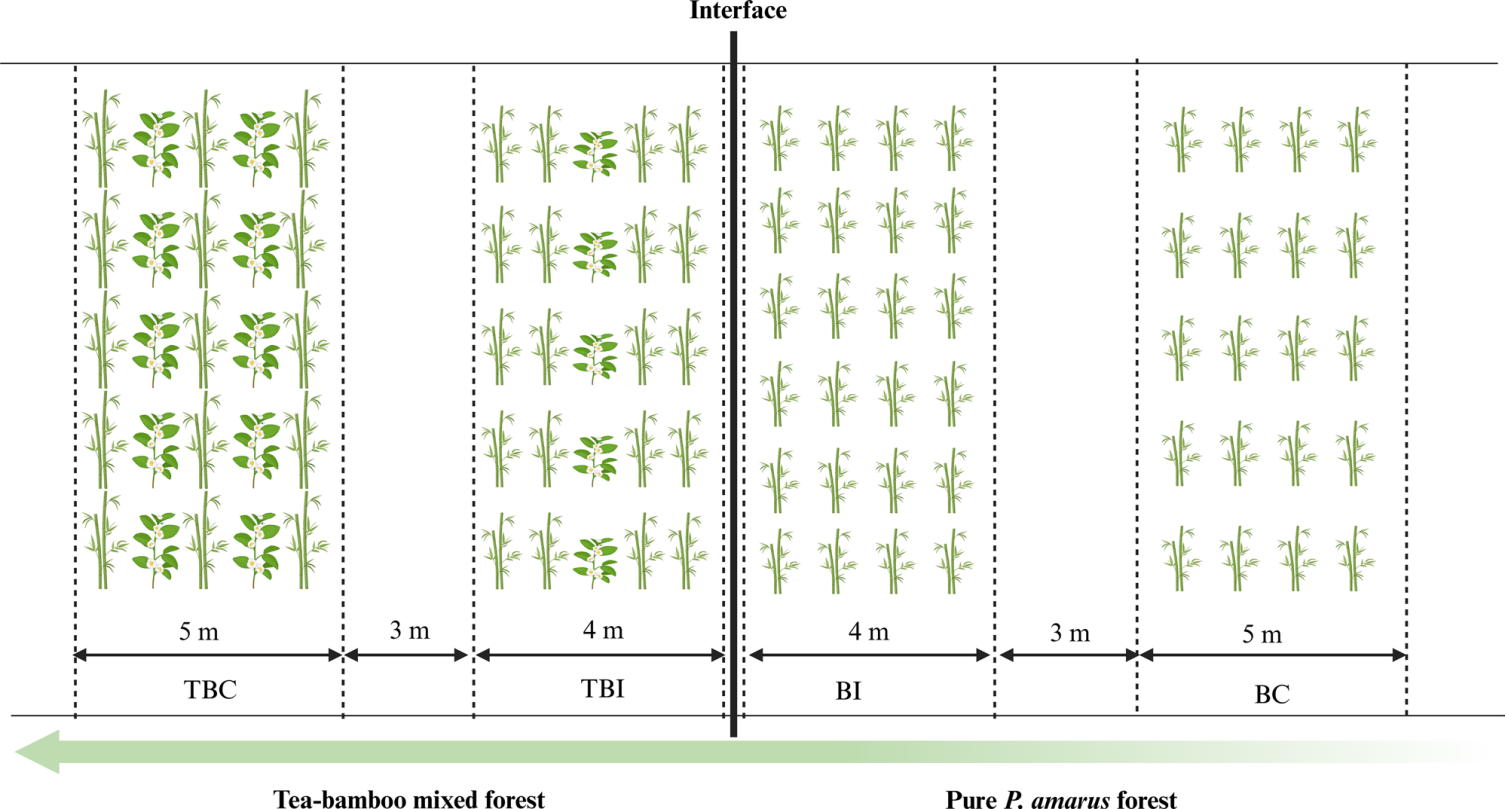
Schematic illustration of sample sites depicting the expansion of pleioblastus amarus into tea plantations. *Note*: BC: bamboo forest center zone, BI: bamboo forest interface zone, TBI: tea-bamboo mixed forest interface zone, TBC: tea-bamboo mixed forest center zone

To assess the effects of bamboo expansion on the quality of bamboo shoots, we conducted a study in two forest types with similar site conditions: pure *P. amarus* forests and tea-bamboo mixed forests. We selected four experimental sites based on the boundary between tea and bamboo areas. On one side of this boundary, we established two sites within the tea-bamboo mixed forest: the tea-bamboo mixed forest interface zone (TBI) and the tea-bamboo mixed forest center zone (TBC), positioned 4 m and 7 m from the boundary, respectively. On the opposite side, within the pure bamboo forest, we set up the bamboo forest interface zone (BI) and the bamboo forest center zone (BC) at the same distances. A schematic illustration of sample sites depicting *P. amarus expansion* into tea plantations was presented in Fig. 1.

Each site measured 20 m in length and approximately 12 m in width of the expanding *P. amarus *area, with at least 3 m of spacing between sites to minimize overlap, except at the interface zones where interactions between bamboo and tea plants were most pronounced. Within each site, we established three 3 × 3 m sampling plots, ensuring a minimum distance of 3 m between adjacent plots. We evaluated the growth characteristics of bamboo and tea plants in each site, including species composition, height, and other relevant parameters (Supplementary Table S1), as well as soil properties (Supplementary Table S2). 

During the bamboo shoot emergence period, 25 complete bamboo shoots was randomly sampled from each 3 × 3 m plot, excavating to a depth of approximately 15 cm. A total of 75 bamboo shoots were selected from each site to assess their appearance traits, ensuring data reliability and consistency across replicates. Each fresh shoot was weighed, and its base diameter and length were recorded and numbered. After removing the sheaths and inedible base portions, we calculated the edible rate (%) using the formula: Edible rate (%) = (Edible portion fresh weight / Total fresh weight) × 100. We also determined height-to-diameter ratio. Additionally, we collected about 2 kg of bamboo shoot samples from each plot, transported them in ice boxes to the laboratory, and processed them. In the lab, we removed the sheaths and inedible bases, homogenized the shoot flesh, and used a portion of the fresh homogenate for analysis of protein, oxalate, tannin, total acid, vitamin C, and free amino acids. Another portion was dried at 70℃ to a constant weight for measurements of fat, soluble sugars, starch, lignin, and cellulose. Each plot was considered as one replicate, with three technical replicates performed for each index measurement.

### Measurement of nutritional indexes

The analysis of soluble sugar and starch content utilized the anthrone colorimetric method [32]. First, 0.2 g of dried sample was mixed with 15 mL of 80% ethanol and heated in a boiling water bath for 10 min. The mixture was then centrifuged at 5,000× g for 10 min. This extraction procedure was repeated three times, and the combined supernatants were adjusted to a final volume of 50 mL. To measure soluble sugar content, 0.2 mL of this extract was mixed with 5 mL of anthrone reagent and heated at 90℃ for 15 min. The absorbance was measured at 620 nm. For starch content, the residue was treated with 10 mL of 30% hydrochloric acid and left overnight. After heating the mixture at 80℃ for 10 min and centrifuging at 4,000× g for 10 min, the supernatant was collected and adjusted to 50 mL. Starch content was quantified by measuring absorbance at 620 nm.

Protein content was assessed following the method described by Xu et al. [5]. 5 g of fresh homogenate was digested with 0.4 g of copper sulfate, 6.0 g of potassium sulfate, and 20 mL of sulfuric acid. The digestion was performed at 420℃ for 1 h until the solution became a clear green. After cooling, 50 mL of water was added, and the sample was analyzed using an automatic Kjeldahl nitrogen analyzer (SKD-2000, Shanghai Peiyu Analytical Instruments Co., Ltd., Shanghai, China).

Fat content was determined using the soxhlet extraction method [33]. The sample was placed in a filter paper tube containing 2 g of ground powder, securely tied with cotton thread, and inserted into the soxhlet extraction apparatus. Extraction was completed when a drop of the extract tested on a glass plate showed no oil spots. The solvent was then recovered, and the remaining solvent in the receiving flask was evaporated in a water bath. The residue was dried at 103℃, cooled in a desiccator for 1 h, and weighed. This process was repeated until a constant weight was achieved, with the weight difference between two consecutive measurements not exceeding 2 mg.

### Analysis of amino acid composition

5.0 g of fresh homogenate was extracted with 60 mL of distilled water in a boiling water bath for 1 h. After cooling to room temperature, the mixture was filtered into a 100 mL volumetric flask, and the residue was washed twice with 20 mL of deionized water. The combined washings and filtrate were adjusted to 100 mL. A 5 mL aliquot of this solution was mixed with 5 mL of 10% sulfosalicylic acid solution and centrifuged at 10,000× g for 15 min at 4℃. The supernatant was diluted to 0.22 mL and analyzed using an amino acid analyzer (L-8900, Hitachi, Japan). Mobile phase preparation followed Fan et al. [32], with a 20 µL injection volume and a flow rate of 0.35 mL·min⁻¹ for the buffer and dansyl chloride.

Amino acid content was categorized as follows: The essential amino acids (EAA) include threonine (Thr), valine (Val), isoleucine (Ile), leucine (Leu), lysine (Lys), methionine (Met), cysteine (Cys), phenylalanine (Phe), and tyrosine (Tyr). The umami amino acids are aspartic acid (Asp) and glutamic acid (Glu). The bitter amino acids include Val, Ile, Leu, Tyr, and Phe. The aromatic amino acids are Phe and Tyr, while the sweet amino acids comprise Thr, glycine (Gly), alanine (Ala), proline (Pro), and serine (Ser) [5].

Amino acid scores (AAS) were calculated according to FAO/WHO recommended standards [34] using the formula:

$$\:ASS=\frac{{A}_{x}}{{A}_{s}}\times\:100$$

where $\:{A}_{x}$ represents the EAA content in the bamboo shoot protein and $\:{A}_{s}$denotes the recommended EAA content values (g·100 g^− 1^): Thr 23, Val 39, lle 30, Leu59, Lys 45, Met + Cys 22, and Phe + Tyr 38.

The essential amino acid index (*EAAI*) was calculated as follows:

$$\:EAAI=\sqrt[\text{n}]{\frac{{\text{T}}_{\text{T}\text{h}\text{r}}}{{\text{S}}_{\text{T}\text{h}\text{r}}}\times\:\frac{{\text{T}}_{\text{v}\text{a}\text{l}}}{{\text{S}}_{\text{v}\text{a}\text{l}}}\dots\:\times\:\frac{{\text{T}}_{\text{P}\text{h}\text{s}+\text{T}\text{y}\text{r}}}{{\text{S}}_{\text{P}\text{h}\text{s}+\text{T}\text{y}\text{r}}}}\times\:100$$

where *n* is the number of EAA compared (*n* = 7 in this study); *T* the EAA content in the bamboo shoot protein; and *S* is the EAA content in egg protein (g·100 g^− 1^). The EAA values for egg protein are: Thr 40, Val 50, lle 40, Leu 70, Lys55, Met + Cys 35, and Phe + Tyr 60.

The nutritional index (*NI*) was determined using the formula:

$$\:NI=\frac{EAAI\times\:PP}{100}$$

where *PP* represents the percentage of protein in the sample.

The closeness degree (*CD*) was calculated as:

$$\:CD=1-C{\sum\:}_{K=1}^{7}\frac{|{a}_{k}-{u}_{ik}|}{|{a}_{k}+{u}_{ik}|}$$

where $\:{a}_{k}$ denotes the EAA content in egg protein, $\:{u}_{ik}$ is the EAA content in bamboo shoot protein, and *C* is a constant (C = 0.09 in this study).

### Determination of flavor substances

Oxalic acid content was determined using high-performance liquid chromatography (HPLC) [35]. 5 g of fresh homogenate was mixed with 30 mL of 0.05% sulfuric acid solution and diluted to 50 mL. A 5 mL aliquot of the extract was centrifuged at 10,000 × g for 10 min at 4℃, and the supernatant was filtered through a 0.22 μm syringe filter. Analysis was performed using a Waters Alliance e2695 HPLC system with an Aminex HPX-87 H column (7.8 × 300 mm). The mobile phase was 0.1% sulfuric acid, with a column temperature of 35℃, the flow rate of 0.60 mL·min^− 1^, and an injection volume of 10 µL. Detection was conducted at 210 nm and 254 nm, using a differential refractive index detector (RID) set to 35℃.

Tannin content was measured using the Folin-Ciocalteu colorimetric method [36]. 5 gof fresh homogenate was mixed with 80 mL of water and heated for 30 min. After filtering, the solution was diluted to 100 mL. A 2 mL aliquot of the extract was centrifuged at 8,000 × g for 4 min. One milliliter of the supernatant was combined with 5 mL of water, 1 mL of sodium tungstate-molybdate solution, and 3 mL of 75 g·L^− 1^ sodium carbonate solution. After standing for 2 h, absorbance was measured at 765 nm.

Total acid content was determined by titration [37]. 200 g of fresh homogenate was dissolved in an equal volume of boiling water, and the filtrate was collected. 50 g of the filtrate was mixed with 60 mL of water and 0.2 mL of 1% phenolphthalein indicator, then titrated with 0.1 mol·L^− 1^ sodium hydroxide solution to a stable red endpoint. The volume of sodium hydroxide used was recorded to calculate the total acid content.

Cellulose and lignin content was measured using sulfuric acid hydrolysis [38]. 100 mg of dried sample was treated with 70 mL of neutral detergent (pH = 7.0) at 100℃ for 40 min, followed by 20 min at 115–121℃. The sample was washed twice with 95% ethanol and absolute ethanol, filtered to pH 6.5-7.0, and vacuum-dried for 20 min. The residue was heated with 70 mL of 2 mol·L^− 1^ hydrochloric acid at 100℃ for 50 min, washed to neutrality, and dried at 80℃ to constant weight (W1). The residue was then treated with 10 mL of 72% sulfuric acid at 20℃ for 4 h, followed by the addition of 90 mL of distilled water and incubation at room temperature overnight. The residue was washed to pH 6.5, dried at 80℃ to constant weight (W2), and burned at 550℃ to constant weight (W3). Cellulose content was calculated as W1 - W2, and lignin content as W2 - W3.

Vitamin C content was determined by HPLC [37]. A 0.15 g freeze-dried sample was suspended in 10 mL of 8% acetic acid-3% meta-phosphoric acid solution and centrifuged at 25 rpm for 5 min, followed by centrifugation at 18,500 × g for 30 min at 4℃. The supernatant was filtered. For ascorbic acid quantification, the filtrate was injected directly. For total vitamin C, the extract was incubated with 10 mM DTT for 30 min to reduce dehydroascorbic acid to ascorbic acid. Separation was performed using an Atlantis T3 column (150 × 4.6 mm ID, 3.5 μm) with a gradient of 0.1% formic acid and methanol: starting at 20% methanol, increasing to 90% over 15 min, maintaining at 90% for 5 min, decreasing back to 20% over 5 min, and holding at 20% for 5 min. The injection volume was 15 µL, the flow rate was 0.5 mL·min^− 1^, and the column temperature was 25℃. Detection was at 254 nm, with quantification based on an external standard curve.

### Data processing

Data were organized using Excel 2010 and analyzed with SPSS 26.0. We performed one-way ANOVA and independent samples t-tests to compare the quality indicators of *P. amarus* shoots across different forest types. Results were reported as means ± standard error, with statistical significance set at α = 0.05. Graphical representations were created using GraphPad Prism 9.5.

To identify the factors influencing changes in bamboo shoot quality during the expansion of *P. amarus* into tea plantations and to explore their interactions, we employed partial least squares structural equation modeling (PLS-SEM) using SmartPLS. This analysis integrated data on the growth characteristics of *P. amarus* and tea plants (Supplementary Table S1) and soil properties (Supplementary Table S2).

## Results

### Changes in appearance and edibility across different sampling sites following the expansion of *P. Amarus* in tea plantations

**Fig. 2:**
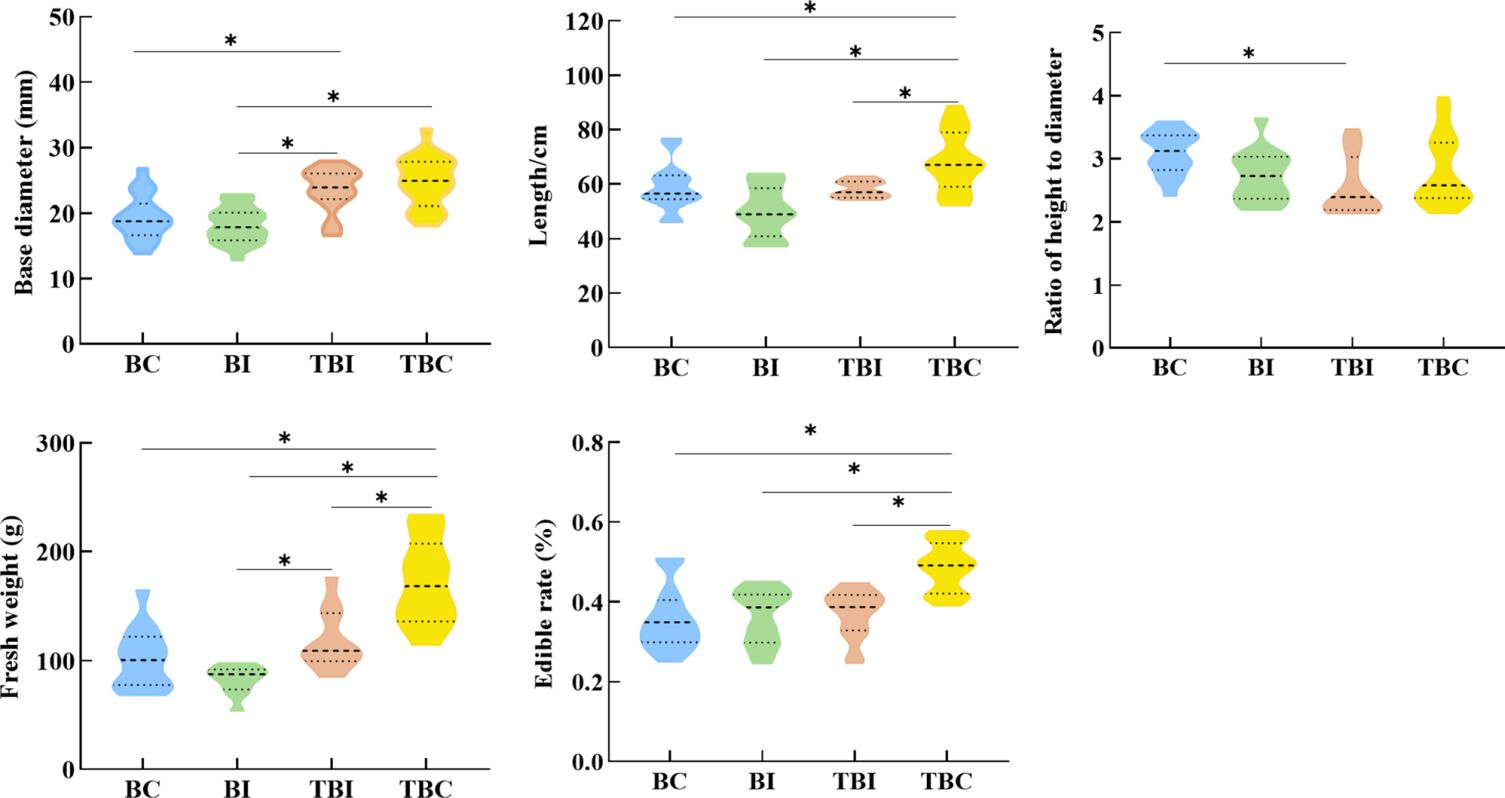
Morphological characteristics and edibility rate changes of *p. amarus* shoots across expansion zones in tea plantations. *Note*: * indicates significant differences between sampling sites (*p* < 0.05). BC: bamboo forest center zone, BI: bamboo forest interface zone, TBI: tea-bamboo mixed forest interface zone, TBC: tea-bamboo mixed forest center zone

The expansion of bamboo forests significantly affected the morphology and edible rate of *P. Amarus* shoots in tea plantations (Fig. 2). As bamboo expanded, the base diameter, length, and fresh weight of the shoots initially decreased, reaching their lowest values at the BI site (18.58 mm, 49.83 cm, and 82.51 g, respectively), before increasing at the TBC site. The TBC site recorded significantly higher values for these indexes compared to the BC, BI, and TBI sites (*p* < 0.05). The height-to-diameter ratio exhibited a similar pattern, with the lowest ratio observed at the TBI site (2.58), which was significantly lower than that of the BC site (*p* < 0.05), but not significantly different from the BI and TBC sites. Moreover, the edible rate of bamboo shoots was significantly higher at the TBC site, being 1.36, 1.34, and 1.31 times greater than at the BC, BI, and TBI sites, respectively. No significant differences in edibility were observed among the BC, BI, and TBI sites.

### Changes in nutritional quality across different sampling sites following the expansion of *P. Amarus* in tea plantations

**Fig. 3:**
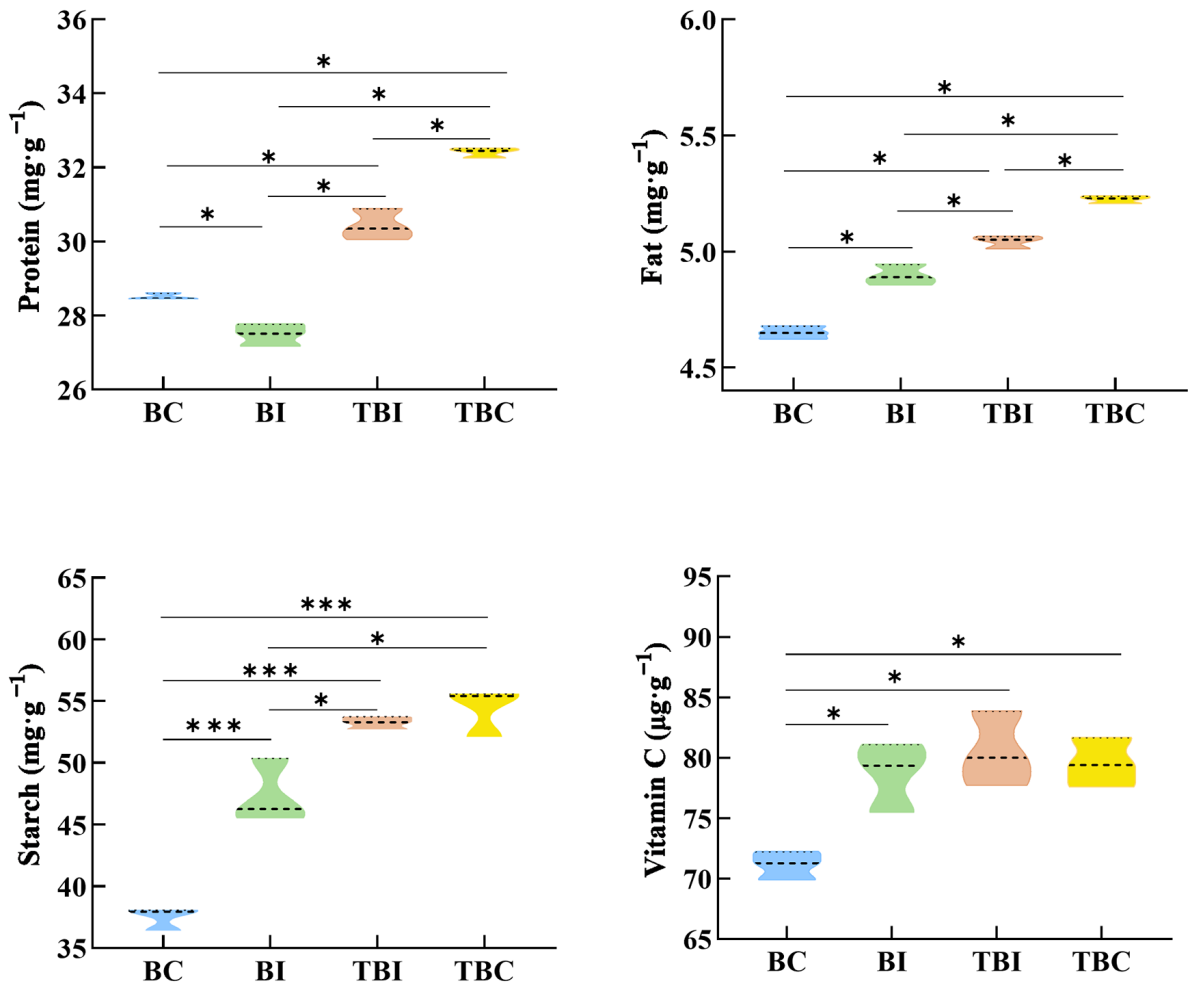
Nutritional changes of *P. amarus* shoots across different expansion zones in tea plantations. *Note*: * and *** indicate significant and highly significant differences between sampling sites (*p* < 0.05). BC: bamboo forest center zone, BI: bamboo forest interface zone, TBI: tea-bamboo mixed forest interface zone, TBC: tea-bamboo mixed forest center zone

The expansion of *P. Amarus* into tea plantations significantly affected the nutritional quality of the bamboo shoots (Fig. 3). Protein and fat content varied significantly among the sites (*p* < 0.05). As bamboo expanded, protein content initially decreased, reaching a minimum of 27.48 mg·g⁻¹ at the BI site, which was 4.92 mg·g⁻¹ lower than at the TBC site. In contrast, fat and starch contents increased, with the lowest values recorded at the BC site (4.65 mg·g⁻¹ and 37.47 mg·g⁻¹, respectively). These values were significantly lower than those at the BI, TBI, and TBC sites (*p* < 0.05), with starch content showing extremely significant differences (*p* < 0.001) compared to the BC site. Vitamin C content increased initially and then stabilized, peaking at 80.54 mg·g⁻¹ at the TBI site. The TBC, TBI, and BI sites had significantly higher Vitamin C levels than the BC site (*p* < 0.05), with no significant differences among the three sites.

**Fig. 4:**
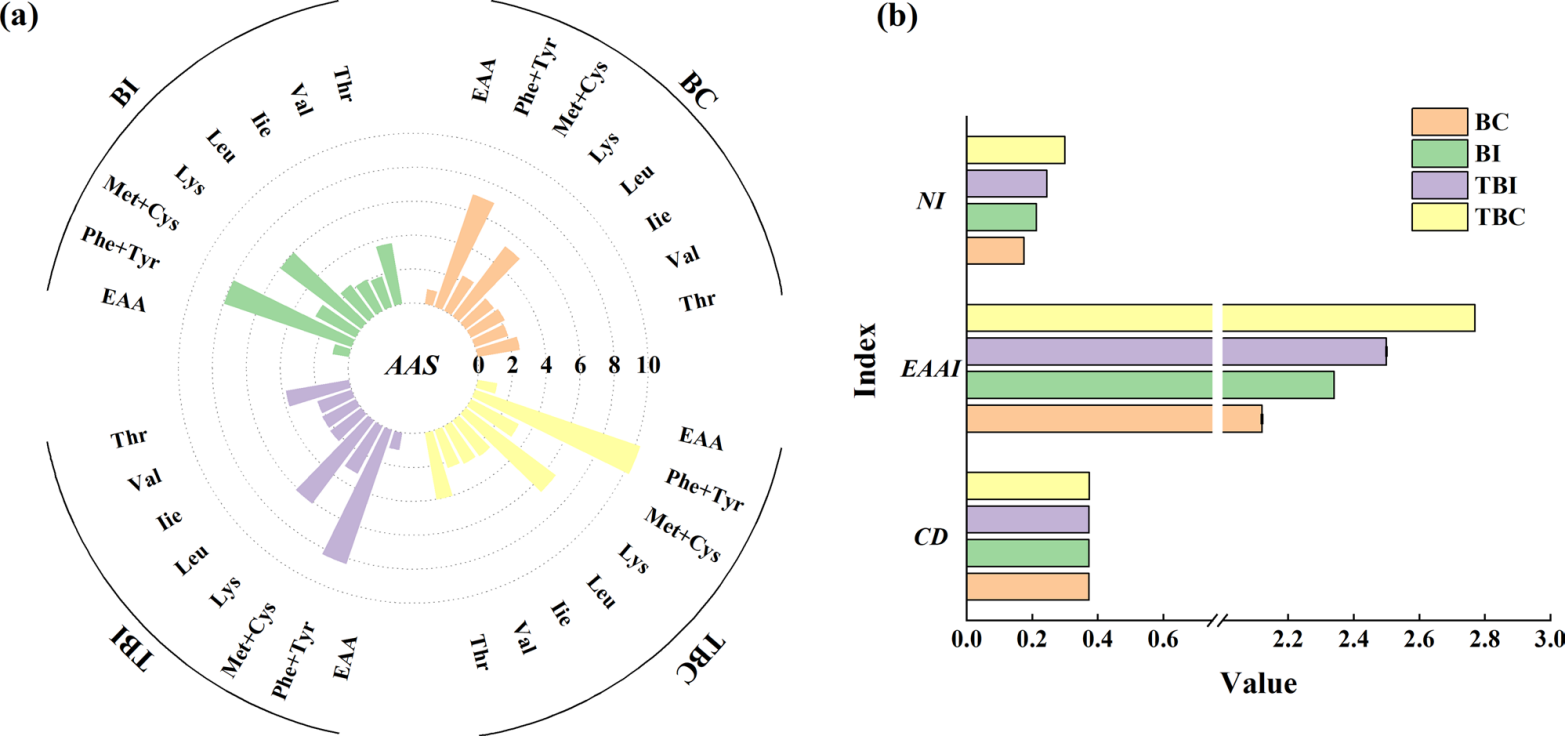
Amino acid scores (**a**) and nutritional index (**b**) of *P. amarus* shoots across different expansion zones in tea plantations. *Note*: AAS: amino acid scores, NI: nutritional index, EAAI: essential amino acid index, and CD: closeness degree. BC: bamboo forest center zone, BI: bamboo forest interface zone, TBI: tea-bamboo mixed forest interface zone, TBC: tea-bamboo mixed forest center zone

Further analysis revealed a gradual increase in *AAS* values of essential amino acids during bamboo expansion, with the highest values observed at the TBC site (Fig. 4). Specifically, Phe + Tyr and Lys showed the highest *AAS* values across all sampling sites, while other essential amino acids remained below FAO/WHO standards, indicating potential nutritional limitations. Despite the relatively consistent *CD* values across sites, *EAAI* and *NI* values increased progressively, reaching their peak at the TBC site, suggesting that bamboo shoots from this site had the highest overall nutritional quality.

### Changes in flavor quality of bamboo shoots across different sampling sites following the expansion of *P. Amarus* in tea plantations

**Fig. 5:**
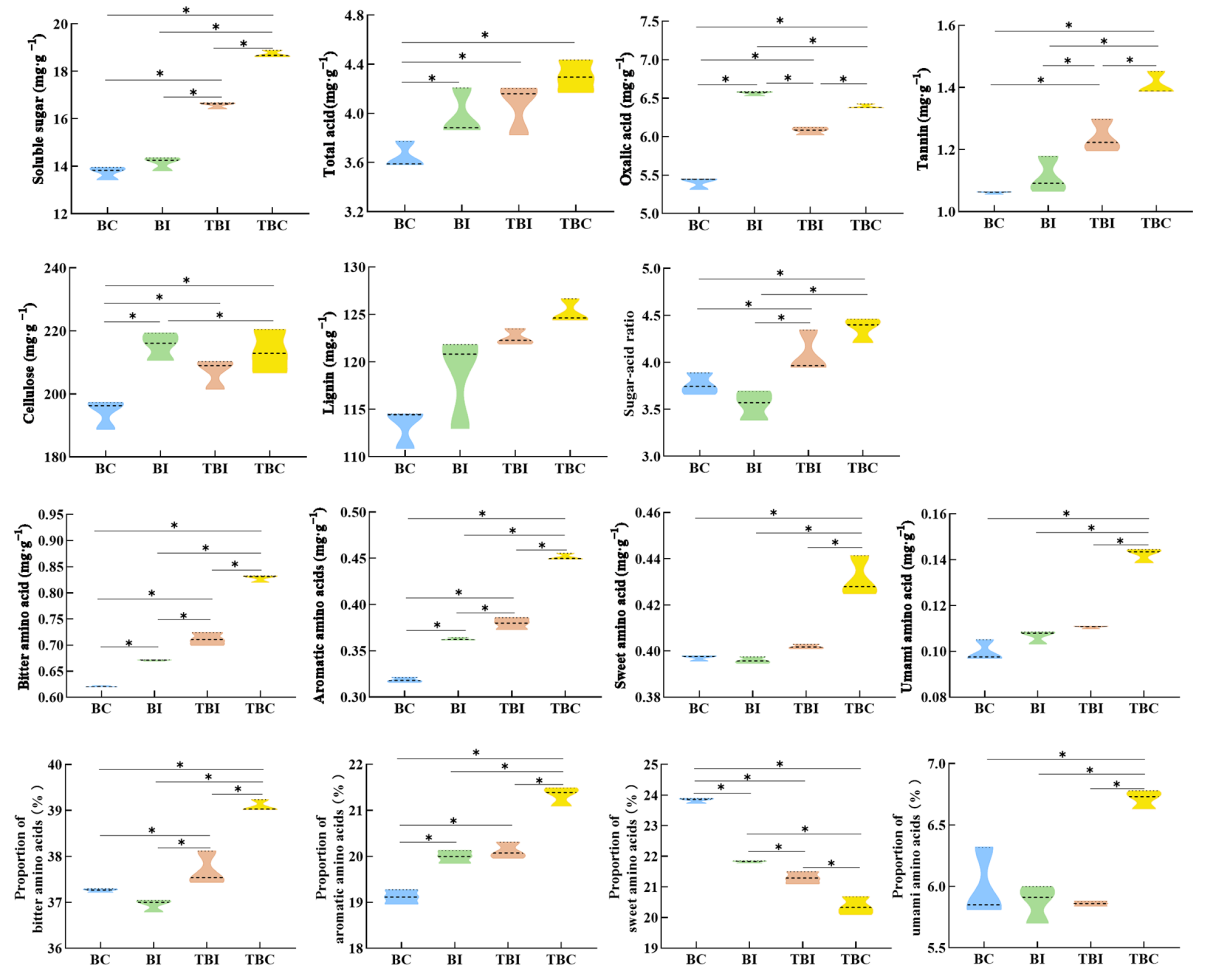
Changes in flavor quality of *P. amarus* shoots across different expansion zones in tea plantations. *Note*: * indicates significant differences between sampling sites (*p* < 0.05). BC: bamboo forest center zone, BI: bamboo forest interface zone, TBI: tea-bamboo mixed forest interface zone, TBC: tea-bamboo mixed forest center zone

The expansion of bamboo into tea plantations significantly altered the flavor quality of *P. Amarus* shoots (Fig. 5). As bamboo expanded, the concentrations of soluble sugars, total acid, tannins, and lignin increased, peaking at 18.72 mg·g⁻¹, 4.27 mg·g⁻¹, 1.41 mg·g⁻¹, and 125.20 mg·g⁻¹, respectively, at the TBC site. These values were 1.36, 1.18, 1.33, and 1.11 times higher than those at the BC site. Oxalic acid and cellulose contents initially rose, reaching peaks at the BI site with levels of 6.57 mg·g⁻¹ and 215.36 mg·g⁻¹, respectively, which were significantly higher than those at the BC site (*p* < 0.05). At the TBI site, oxalic acid and cellulose levels declined compared to the BI and TBC sites, though these changes were not statistically significant. The sugar-to-acid ratio decreased initially and then increased, reaching its lowest point at the BI site before rising as expansion continued. Regarding flavor amino acids, the proportions of umami, aromatic, and bitter amino acids increased, while sweet amino acids decreased. At the TBC site, the proportion of sweet amino acids reached its lowest value of 20.37%.

### Analysis of driving factors in bamboo shoot quality formation during *P. Amarus *expansion into tea plantations

**Fig. 6:**
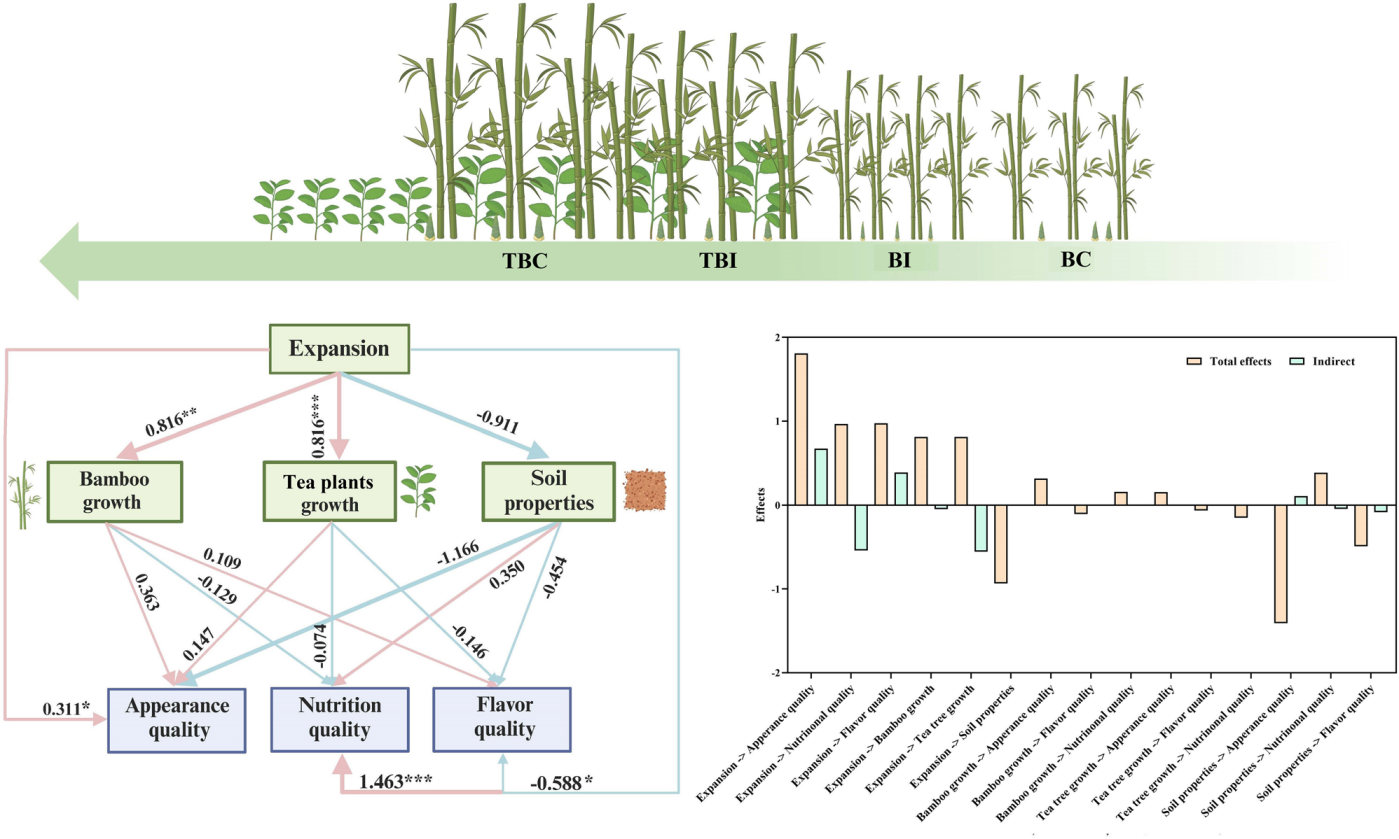
Driving factors affecting bamboo shoot quality during the expansion of *p. amarus* expansion within tea plantations. *Note*: The left panel depicts the relationships between driving factors, with red and blue arrows representing positive and negative effects, respectively. Arrow width denotes standardized path coefficients, with thicker arrows indicating higher coefficients. Numbers adjacent to arrows indicate standardized path coefficients. Significance levels are denoted as *p < 0.05, **p < 0.01, ***p < 0.001. The right panel shows the total and indirect effects of these factors on bamboo shoot quality during the expansion. BC: bamboo forest center zone, BI: bamboo forest interface zone, TBI: tea-bamboo mixed forest interface zone, TBC: tea-bamboo mixed forest center zone

PLS-SEM analysis (Fig. 6) revealed that bamboo expansion into tea plantations had both positive and negative effects on bamboo shoot quality. Bamboo expansion had a highly significant positive impact on the appearance and nutritional quality of the shoots (*p* < 0.01), with a more pronounced direct effect on nutritional quality. However, it had a significant negative effect on flavor quality (*p* < 0.05). Additionally, bamboo expansion positively affected bamboo growth characteristics (*p* < 0.01) but had a negative effect on soil factors.

The total effect of soil factors was greater than that of bamboo and tea plants growth characteristics, influencing shoot quality directly and indirectly. Among soil factors, total phosphorus (TP) and total potassium (TK) had the highest impacts. Bamboo and tea plants growth characteristics and soil properties both had direct positive and negative effects on shoot quality, although the differences were not significant. Among bamboo growth characteristics, diameter at breast height (DBH) and density were the most significant (*p* < 0.001). For tea plants growth characteristics, tree height and crown width were the most influential (*p* < 0.001) (Supplementary Table S3). Shoot length and fresh weight were identified as important factors influencing the appearance quality of bamboo shoots. Fat, starch, total amino acids, essential amino acids, and lignin were key factors affecting nutritional quality, while flavor quality was primarily related to soluble sugars, umami amino acids, and bitter amino acids (Supplementary Table S3).

## Discussion

### Formation of bamboo shoot appearance quality and key driving factors during *P. Amarus *expansion into tea plantations

This study found that during the expansion of *P. Amarus* into tea plantations, the base diameter, length, and fresh weight of bamboo shoots generally increased, indicating an improvement in appearance quality. However, at the BI site, these parameters decreased, likely because bamboo had not yet occupied a favorable spatial position within the tea plantation. Limited light and nutrient availability at this stage constrained shoot growth. As expansion progressed, the TBI and TBC sites exhibited improvements, with bamboo gradually occupying more advantageous space. This spatial shift improved light conditions, and nutrient availability [39, 40], promoting shoot growth and development. At the TBC site, the base diameter, length, fresh weight, and edible rate of bamboo shoots were significantly higher than those at the BC site. This suggests that the bamboo-tea interactive environment formed after expansion better supports the development of high-quality shoot appearance, consistent with the PLS-SEM analysis results.

The PLS-SEM analysis indicated that bamboo growth characteristics positively affected appearance quality, with bamboo diameter and density being key driving factors. At the BC site, the smaller bamboo size and higher density intensified intraspecific competition, limiting access to light, water, and space [41], ect., and thereby constraining shoot growth. In contrast, at the TBC site, optimal bamboo growth and lower density improved shoot appearance, emphasizing interactively an optimized bamboo stand structure for enhancing shoot quality.

Additionally, PLS-SEM analysis revealed that the expansion of *P. Amarus* negatively impacted soil properties, leading to gradual soil degradation. However, despite the decline in soil quality, the increase in bamboo shoot size indicated a significant indirect effect of soil properties on the appearance quality of the shoots. Soil characteristics influenced shoot growth and appearance by affecting root development, enhancing photosynthetic efficiency, and improving water utilization [42, 43]. At the TBC expansion phase, the improved bambooforest structure likely enhanced soil nutrient utilization, supported root development, and facilitated resource acquisition, further improving shoot appearance quality.

### Nutritional quality and key driving factors of bamboo shoots during the expansion of *P. Amarus *into tea plantations

This study found that as *P. amarus* expanded into tea plantations, there was a progressive increase in the protein, fat, starch, and vitamin C contents of bamboo shoots. Concurrently, the indexes of *AAS*, *EAAI*, and *NI* improved, indicating a significant enhancement in the nutritional value and quality of the bamboo shoots. These findings align with the PLS-SEM analysis, which highlighted that bamboo expansion positively affects the nutritional quality. In the TBC plot, bamboo shoots exhibited superior levels of essential amino acids and overall nutritional components, attributable to improved growth conditions, enhanced nutrient uptake efficiency [2], and the increased adaptability of *P. amarus* to the new environment. The increases in protein, fat, and vitamin C contents demonstrate the notable phenotypic plasticity of *P. amarus*, enabling it to adjust its physiological metabolism in response to environmental changes [44]. This adaptability not only benefits the expansion of *P. amarus* but also enhances the nutritional quality and market value of its shoots.

PLS-SEM analysis further revealed that soil properties significantly influenced the nutritional quality of bamboo shoots. Specifically, soil TP and TK levels played critical roles in shoot growth and nutritional development [6, 45]. Despite TP and TK levels were lower in the TBC plot, *P. amarus* likely employed efficient nutrient absorption and allocation strategies to enhance bamboo shoot development, thereby improving their nutritional quality. Thus, the ability of *P. amarus* to adapt its metabolism and resource acquisition strategies to changing environmental conditions, combined with improvements in soil nutrients, plays a key role to enhancing the nutritional quality of bamboo shoots.

### Flavor quality and key driving factors of bamboo shoots during the expansion of *P. Amarus *into tea plantations

This study found a progressive increase in soluble sugar content and the sugar-to-acid ratio in bamboo shoots as *P. amarus* expanded into tea plantations, with the highest levels observed in the TBC plot. These findings suggest that higher sugar content and improved sugar-to-acid ratios can enhance the flavor and palatability of bamboo shoots, potentially boosting growth rates and environmental adaptability [5, 32, 46]. This indicates that *P. amarus* shows notable adaptability and competitiveness in tea plantation environments, demonstrating its potential to produce high-quality bamboo shoots.

The increase in flavor amino acids during the expansion further reflects an improvement in the flavor quality of the bamboo shoots. This enhancement likely stems from better growth conditions, such as optimized light and nutrient availability, which promote the synthesis and accumulation of flavor amino acids [47, 48]. Although the absolute content of sweet amino acids increased, their relative proportion decreased. This suggests that the increase in other amino acids (e.g., bitter, aromatic, and umami) was more substantial, altering the overall flavor balance of the bamboo shoots.

However, despite the rise in certain flavor compounds, higher levels of tannins, total acids, and lignin may negatively impact the bamboo shoots’ palatability, making them potentially more bitter, acidic, and coarse [32, 49, 50]. PLS-SEM analysis revealed a negative overall effect of the expansion on these flavor compounds. Although *P. amarus* benefits from improved light and nutrient conditions in tea plantations, negative factors such as competition with tea plants and decreased soil nutrients may counteract these benefits. Therefore, while the expansion of *P. amarus* can improve the flavor profile of bamboo shoots, optimizing management and cultivation practices is essential to mitigate these negative factors and further enhance the overall flavor quality of the bamboo shoots.

## Conclusions

The expansion of *P. amarus* into tea plantations significantly improved the appearance and nutritional quality of bamboo shoots, particularly in the TBC plot, where increased in size, edibility, and nutrient content were observed. Protein and amino acids levels also showed notable enhancements. The expansion led to higher soluble sugar content and flavor amino acids, which positively impacted the flavor profile. However, elevated levels of oxalic acid, tannins, and lignin contributed to greater bitterness and roughness, negatively affecting palatability. PLS-SEM analysis revealed that while the expansion positively influenced the appearance and nutritional quality of the bamboo shoots, it had a detrimental effect on flavor quality. To mitigate these negative effects on palatability, future management strategies should prioritize optimizing soil conditions and nutrient balance. These findings offer valuable insights into bamboo shoot production, suggesting that targeted adjustments in soil management could enhance overall quality, particularly in flavor and texture, as *P. amarus* continues to expand into tea plantations.

## Electronic supplementary material

Below is the link to the electronic supplementary material.

Supplementary Material 1: Table S1. Basic growth conditions of bamboo and tea plants across various sampling sites after the expansion of p. amarus within tea plantations. note: different lowercase letters indicate significant differences at the 0.05 level. bc: bamboo forest center zone, bi: bamboo forest interface zone, tbi: tea-bamboo mixed forest interface zone, tbc: tea-bamboo mixed forest center zone. Table S2 Soil properties across various sampling sites after the expansion of p. amarus within tea plantations. note different lowercase letters indicate significant differences at the 0.05 level. OM: organic matter, OC: organic carbon, TN: total nitrogen, TP: total phosphorus, TK: total potassium, HN: hydrolyzable nitrogen, AP: available phosphorus, and AK: available potassium. BC: bamboo forest center zone, BI: bamboo forest interface zone, TBI: tea-bamboo mixed forest interface zone, TBC: tea-bamboo mixed forest center zone. Table S3 driving factors and path coefficients influencing the quality formation of bamboo shoots after the expansion of p. amarus within tea plantations. note om: organic matter, OC: organic carbon, TN: total nitrogen, TP: total phosphorus, TK: total potassium, HN: hydrolyzable nitrogen, AP: available phosphorus, AK: available potassium. BS: bamboo shoot base diameter, BL: bamboo shoot length, BW: bamboo shoot fresh weight, BE: bamboo shoot edible rate, BHD: bamboo shoot height-to-diameter ratio, SP: protein, ST: starch, Vit: vitamin C, Cel: cellulose, Lin: lignin, TAA: total amino acid, EAA: essential amino acid, TAN: tannin, OA: oxalic acid, TA: total acid, SS: soluble sugar, SS/TA: soluble sugar/ total acid, UAA: umami amino acid, SAA: sweet amino acid, BAA: bitter amino acid, and AAA: aromatic amino acid.

## Data Availability

The datasets supporting the conclusions of this article are included within the article and its supplementary files” by selecting.
