# Supplementary material for: Expansion of Pleioblastus amarus in tea plantations significantly enhances the appearance and nutritional composition of bamboo shoots but adversely affects palatability
Source: BMC Plant Biol. 2024 Dec 3;24:1161. doi: 10.1186/s12870-024-05856-1 (PMC11613805; doi:10.1186/s12870-024-05856-1)
Supplement: Supplementary file 1 — Supplementary Material 1: Table S1. Basic growth conditions of bamboo and tea plants across various sampling sites after the expansion of p. amarus within tea plantations. note: different lowercase letters indicate significant differences at the 0.05 level. bc: bamboo forest center zone, bi: bamboo forest interface zone, tbi: tea-bamboo mixed forest interface zone, tbc: tea-bamboo mixed forest center zone. Table S2 Soil properties across various sampling sites after the expansion of p. amarus within tea plantations. note different lowercase letters indicate significant differences at the 0.05 level. OM: organic matter, OC: organic carbon, TN: total nitrogen, TP: total phosphorus, TK: total potassium, HN: hydrolyzable nitrogen, AP: available phosphorus, and AK: available potassium. BC: bamboo forest center zone, BI: bamboo forest interface zone, TBI: tea-bamboo mixed forest interface zone, TBC: tea-bamboo mixed forest center zone. Table S3 driving factors and path coefficients influencing the quality formation of bamboo shoots after the expansion of p. amarus within tea plantations. note om: organic matter, OC: organic carbon, TN: total nitrogen, TP: total phosphorus, TK: total potassium, HN: hydrolyzable nitrogen, AP: available phosphorus, AK: available potassium. BS: bamboo shoot base diameter, BL: bamboo shoot length, BW: bamboo shoot fresh weight, BE: bamboo shoot edible rate, BHD: bamboo shoot height-to-diameter ratio, SP: protein, ST: starch, Vit: vitamin C, Cel: cellulose, Lin: lignin, TAA: total amino acid, EAA: essential amino acid, TAN: tannin, OA: oxalic acid, TA: total acid, SS: soluble sugar, SS/TA: soluble sugar/ total acid, UAA: umami amino acid, SAA: sweet amino acid, BAA: bitter amino acid, and AAA: aromatic amino acid. [file 12870_2024_5856_MOESM1_ESM.docx]

***Supplementary materials***

*Table S1. Basic Growth Conditions of Bamboo and Tea* *Plants across Various Sampling Sites after the Expansion of Pleioblastus Amarus within Tea Plantations.*

| Sampling sites | *P. amarus* | | | Tea plants | | |  | Elevation/m | Aspect | Slope/° |
| --- | --- | --- | --- | --- | --- | --- | --- | --- | --- | --- |
|  | Density/(clum·hm^-2^) | Mean DBH/mm | Mean height/m | Mean height/m | Crown/m | Number of forks/individual | Band spacing/m |  |  |  |
| BC | 41 131.67±5 124.53a | 8.83±0.06b | 14.58±0.20b | / | / | / | / | 230-280 | West | 30~35° |
| BI | 48 357.50±13 277.32a | 9.53±0.75b | 14.53±0.68b | / | / | / | / | 230-280 | West | 30~35° |
| TBI | 46 273.13±9 442.01a | 13.82±0.87a | 21.01±1.35a | 2.10±0.16a | 1.14±0.38a | 8.00±1.55a | 1.40±0.20a | 230-280 | West | 30~35° |
| TBC | 41 131.67±8 073.954a | 14.91±0.47a | 22.37±0.72a | 1.52±0.10b | 0.98±0.08a | 12.33±2.09a | 1.80±0.01a | 230-280 | West | 30~35° |

*Note: Different lowercase letters indicate significant differences at the 0.05 level. BC: bamboo forest center zone, BI: bamboo forest interface zone, TBI: tea-bamboo mixed forest interface zone, TBC: tea-bamboo mixed forest center zone.*

*Table S2 Soil* *Properties across Various Sampling Sites after the Expansion of P. Amarus within Tea Plantations.*

| Sampling Sites | pH | OM  /(g·kg^-1^) | OC  /(g·kg^-1^) | TN  /(g·kg^-1^) | TP  /(g·kg^-1^) | TK  /(g·kg^-1^) | HN  /(mg·kg^-1^) | AP  /(mg·kg^-1^) | AK  /(mg·kg^-1^) |
| --- | --- | --- | --- | --- | --- | --- | --- | --- | --- |
| BC | 4.52±0.01b | 37.97±0.81ab | 22.03±0.47ab | 1.85±0.05ab | 0.54±0.02a | 26.03±0.87a | 145.80±6.11a | 1.73±0.21a | 181.38±2.18b |
| BI | 4.61±0.01a | 39.83±1.02a | 23.10±0.59a | 1.93±0.05a | 0.55±0.01a | 25.28±0.74a | 146.43±10.75a | 2.48±0.32a | 192.63±0.13ab |
| TBI | 4.66±0.01a | 40.30±0.74a | 23.38±0.43a | 2.01±0.03a | 0.55±0.01a | 23.95±1.19a | 154.46±10.71a | 3.20±0.53a | 201.72±5.93a |
| TBC | 4.61±0.01a | 35.60±1.19ab | 20.65±0.69ab | 1.70±0.07bc | 0.47±0.07b | 18.33±0.36b | 157.95±6.54a | 2.67±0.31a | 185.94±0.17b |

*Note: Different lowercase letters indicate significant differences at the 0.05 level. OM: organic matter, OC: organic carbon, TN: total nitrogen, TP:* *total phosphorus,* *TK: total potassium, HN: hydrolyzable nitrogen, AP: available phosphorus, and AK: available potassium. BC: bamboo forest center zone, BI: bamboo forest interface zone, TBI: tea-bamboo mixed forest interface zone, TBC: tea-bamboo mixed forest center zone.*

*Table S3 Driving Factors and Path Coefficients Influencing the Quality Formation of Bamboo Shoots after the Expansion of P. Amarus within Tea Plantations.*

| Path | Outer loadings | *p* values |
| --- | --- | --- |
| pH <- Soil properties | -0.356 | 0.482 |
| OM <- Soil properties | 0.603 | 0.082 |
| OC <- Soil properties | 0.603 | 0.082 |
| TN <- Soil properties | 0.576 | 0.084 |
| TP <- Soil properties | 0.875 | 0.182 |
| TK <- Soil properties | 0.931 | 0.225 |
| AK <- Soil properties | -0.052 | 0.877 |
| AP <- Soil properties | -0.467 | 0.419 |
| HN <- Soil properties | -0.226 | 0.597 |
| Density <- Bamboo growth | -0.646 | 0.002 |
| DBH <- Bamboo growth | 0.984 | 0.000 |
| Height <- Bamboo growth | 0.956 | 0.000 |
| Tree crown <- Tea plants growth | 0.994 | 0.000 |
| Tree height <- Tea plants growth | 0.995 | 0.000 |
| BS <- Apperance | 0.873 | 0.000 |
| BL <- Apperance | 0.909 | 0.000 |
| BW <- Apperance | 0.984 | 0.000 |
| BE <- Apperance | 0.692 | 0.040 |
| BHD <- Apperance | -0.512 | 0.113 |
| SP <- Nutritional quality | 0.793 | 0.000 |
| Fat <- Nutritional quality | 0.984 | 0.000 |
| ST <- Nutritional quality | 0.960 | 0.000 |
| Vit <- Nutritional quality | 0.853 | 0.000 |
| Cel <- Nutritional quality | 0.749 | 0.000 |
| Lin <- Nutritional quality | 0.946 | 0.000 |
| TAA <- Nutritional quality | 0.944 | 0.000 |
| EAA <- Nutritional quality | 0.969 | 0.000 |
| TAN <- Flavor quality | 0.974 | 0.000 |
| OA <- Flavor quality | 0.540 | 0.029 |
| TA <- Flavor quality | 0.889 | 0.000 |
| SS <- Flavor quality | 0.981 | 0.000 |
| SS/TA <- Flavor quality | 0.829 | 0.000 |
| UAA <- Flavor quality | 0.964 | 0.000 |
| SAA <- Flavor quality | 0.910 | 0.000 |
| BAA <- Flavor quality | 0.991 | 0.000 |
| AAA <- Flavor quality | 0.982 | 0.000 |

*Note: OM: organic matter, OC: organic carbon, TN: total nitrogen, TP:* *total phosphorus,* *TK: total potassium, HN: hydrolyzable nitrogen, AP: available phosphorus, AK: available potassium. BS: bamboo shoot base diameter, BL: bamboo shoot length, BW: bamboo shoot fresh weight, BE: bamboo shoot edible rate, BHD: bamboo shoot height-to-diameter ratio, SP: protein, ST: starch, Vit: vitamin C, Cel: cellulose, Lin: lignin, TAA: total amino acid, EAA: essential amino acid, TAN: tannin, OA: oxalic acid, TA: total acid, SS: soluble sugar, SS/TA: soluble sugar/ total acid, UAA: umami amino acid, SAA: sweet amino acid, BAA: bitter amino acid, and AAA: aromatic amino acid.*
